# Programmed death receptor-1/programmed death-ligand 1 inhibitors: Clinical progress and biomarker exploration in gastric cancer

**DOI:** 10.1016/j.heliyon.2024.e38710

**Published:** 2024-10-04

**Authors:** Jin Shi, Xudong Song, Zihao Gao, Dezhu Dai, Fan Ding, Xu Wu, Wufei Dai, Guoquan Tao

**Affiliations:** a Department of Gastrointestinal Surgery, The Affiliated Huaian No. 1 People's Hospital of Nanjing Medical University, Huai'an, Jiangsu, 223300, China; b Department of Pediatric Surgery, University Children's Hospital Basel, 4031, Basel, Switzerland; c Department of Clinical Research, University of Basel, 4031, Basel, Switzerland; d Department of Vascular, Huaian Hospital Affiliated to Xuzhou Medical University, Huai'an, Jiangsu, 223300, China; e Department of Plastic and Reconstructive Surgery, Shanghai Ninth People’s Hospital, Shanghai Jiao Tong University School of Medicine, Shanghai 200011, China

**Keywords:** Gastric cancer, Immunotherapy, Programmed death receptor-1, Programmed death-ligand 1, Immune checkpoint inhibitors

## Abstract

Gastric cancer is one of the most common malignant tumours, with limited treatment options and poor prognosis in its advanced stages. In recent years, breakthroughs in tumour immunotherapy have led to immune checkpoint inhibitors becoming a new class of clinical oncology drugs. Programmed death receptor-1 (PD-1) and programmed death-ligand 1 (PD-L1) play significant roles in inhibiting T cell responses and tumour immune escape. PD-1/PD-L1 inhibitors can significantly improve the prognosis of patients with advanced gastric cancer. Moreover, the combination of administering PD-1/PD-L1 inhibitors along with chemotherapy, radiotherapy, targeted therapy, and other immunotherapies may further enhance therapeutic efficacy. However, some scientific issues need to be urgently resolved in the immunotherapy of gastric cancer, including the suboptimal efficacy of PD-1/PD-L1 inhibitor monotherapy, high incidence of immune-related adverse events, and the absence of definitive biomarkers for effectively screening treatment-sensitive populations. This article reviews the mechanism of action, therapeutic advances, adverse effects, and putative predictive biomarkers of PD-1/PD-L1 inhibitors in the treatment of advanced gastric cancer.

## 1 Introduction

Gastric cancer (GC) ranks as the third most common cause of cancer-related deaths. Despite improvements in diagnosis rates, survival times, and quality of life, most patients with GC are diagnosed at locally advanced or late stages of the disease with poor prognosis [1,2]. The present treatment situation for patients with advanced GC is not optimistic, with traditional surgery and chemotherapy reaching a plateau in effectiveness, and targeted therapy in GC not progressing smoothly, resulting in very limited treatment options.

Recently, the emergence of immunotherapy has introduced a new ray of hope for the treatment of advanced GC. Immune checkpoint proteins programmed death-1 (PD-1) and its ligand programmed death-ligand 1 (PD-L1) have emerged in the treatment of advanced GC, becoming a hot topic of research domestically in China and internationally [3,4]. At present, a range of immune checkpoint inhibitors, prominently represented by PD-1/PD-L1 inhibitors, have gained approval for treating various tumours, including non-small cell lung cancer, GC, liver cancer, and colorectal cancer [5]. The PD-1 and PD-L1 pathways perform pivotal functions in the modulation of the immune response. PD-1 serves as an inhibitory co-receptor on the surface of T lymphocytes, and PD-L1 acts as a ligand on the surface of various tumour cells. By inhibiting the PD-1/PD-L1 signalling pathway, tumour cells can evade the host immune system. Therefore, the application of PD-1/PD-L1 inhibitors can reinstate the immune activity of T cells in response to tumours, thereby blocking tumour immune escape.

However, not every patient derives benefit from monotherapy with PD-1/PD-L1 antibodies. Although PD-1/PD-L1 antibodies can improve the objective response rate and quality of life of patients, some patients do not respond to these treatments [6]. This indicates that single-agent immunotherapy may have limited effects in certain cases, making combination therapy strategies increasingly a focus of research. Such integrated therapy strategies aim to act on cancer cells through different pathways simultaneously, maximising therapeutic effects and reducing the risk of drug resistance. Although these combination therapy studies are still in the early stages, the accumulated data and integrated application of different treatment modalities can provide more effective and personalised treatment options for patients with cancer.

Cancer immunotherapy has unique toxicities, primarily due to the complexity of its mechanism of action and the substantial activation of the immune system of the patient [7]. In the process of acting on patients with GC, PD-1/PD-L1 inhibitors usually activate or enhance the immune system of the patient to recognise and attack cancer cells, but this process can cause very intense inflammatory responses, leading to a series of treatment-related adverse events. Therefore, while focusing on the efficacy of immunotherapy, the adverse reactions of immunotherapy also need to be seriously considered.

In an effort to enhance the efficacy of PD-1/PD-L1 treatment for GC, researchers are diligently working to identify biomarkers that can reliably predict patient responses to PD-1/PD-L1 antibody therapy. The discovery and application of predictive biomarkers will help to more precisely select suitable immunotherapies and identify patient subgroups most likely to benefit from PD-1/PD-L1 inhibitor therapy. This exploration delves into predictive biomarkers in PD-1/PD-L1 inhibitor therapy, aiming to furnish more substantial evidence to support personalised treatment strategies.

As researchers delve deeper into the complex world of cancer therapy, the emergence of immunotherapy has become a transformative force, offering new dimensions to our methods of combating malignant tumours. In the field of GC, a malignancy with significant global impact, PD-1/PD-L1 inhibitors have become central to the search for effective treatment strategies. In this review article, we systematically evaluate recent advancements in utilizing PD-1/PD-L1 inhibitors within the context of restorative therapies. Distinguishing itself from prior reviews, this article places a particular emphasis on clinical research data, offering a comprehensive overview of the efficacy, safety, and potential biomarkers associated with PD-1/PD-L1 inhibitors in these therapeutic settings. By conducting an in-depth analysis of extensive clinical trial data, this review seeks to provide clinicians and researchers with the most current insights, thereby serving as a reliable resource for guiding patient care in restorative therapy.

## 2 Anti-PD-1/PD-L1 immune suppression mechanism

Under normal physiological conditions, PD-1 maintains immune balance and self-tolerance by binding with PD-L1, preventing autoimmune cells (T lymphocytes) from excessively attacking host tissues, thereby regulating chronic inflammation and tissue damage caused by autoimmune diseases [8,9]. However, in tumour cells, the upregulation of PD-1, upon binding with PD-L1, leads to the phosphorylation of its intracellular immunoreceptor tyrosine-based switch motif (ITSM) by Src family kinases (SFKs). The phosphorylated PD-1 ITSM domain recruits SH2 domain-containing phosphatases (SHP-2) that dephosphorylate key signalling molecules involved in T cell activation, including SFKs, Syk, and phosphoinositide 3-kinase (PI3K). The phosphatases recruited by PD-1 dephosphorylate Syk, PI3K, and other signalling molecules, effectively inhibiting TCR signal transduction, reducing cytokine production, and ultimately suppressing T cell activation and proliferation [10,11]. The PD-1/PD-L1 interaction provides inhibitory signals during the effector phase of T cell responses, suppressing T cell functions through multiple mechanisms. Specifically, the PD-1/PD-L1 signalling pathway can inhibit the proliferation and survival of CD8^+^ T cells, weaken their effector functions, and induce Fas-mediated T cell apoptosis [12]. This inhibitory mechanism is a crucial strategy by which tumour cells evade immune surveillance.

**Fig. 1:**
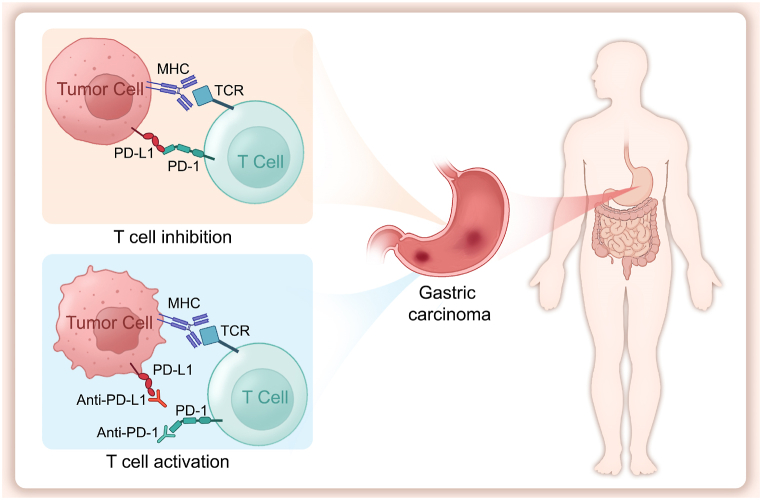
Model of the interaction between programmed death-1 (PD-1) and programmed death-ligand 1 (PD-L1) in gastric cancer. PD-1 combines with its ligand PD-L1, resulting in T-cell inhibition and immune evasion. PD-1/PD-L1 inhibitors function by binding to either PD-1 or PD-L1, thereby blocking the PD-1:PD-L1 interaction, which allows T cells to regain their activity. The T cell receptor (TCR) recognizes antigens presented by the major histocompatibility complex (MHC), leading to the activation of T cells, which is the first step in initiating an immune response.

Recent advancements in therapies utilizing PD-1/PD-L1 inhibitors have shown significant benefits for patients with advanced-stage tumours. As shown in Fig. 1, leveraging the immunomodulatory functions of PD-1 and PD-L1, these inhibitors have become integral in tumour immunotherapy. PD-1 and PD-L1 inhibitors (such as Nivolumab, Pembrolizumab, and Atezolizumab) block the interaction between PD-1 and PD-L1, disrupting this negative regulatory mechanism and reactivating T cell immune responses against tumour cells. These inhibitors lift the functional inhibition of T cells, restoring their proliferative capacity and effector functions, thereby enhancing the cytotoxicity of T cells against tumour cells. By boosting antitumour immune responses, this mechanism significantly improves the treatment efficacy of various malignancies, demonstrating broad clinical application prospects.

## 3 PD-L1/PD-1 inhibitor therapy

### 3.1 PD-L1/PD-1 inhibitor monotherapy in GC

PD-1/PD-L1 immune checkpoint inhibitor (ICI) clinical trials in monotherapy or combination therapy for GC have shown significant efficacy and robust safety, providing a favourable scientific basis for their clinical application (Table 1). The PD-1/PD-L1 inhibitors currently used in clinical or ongoing clinical trials include nivolumab, pembrolizumab, and avelumab. Based on the results of the ATTRACTION-2 clinical trial, the median survival and 3-year survival rate values of the nivolumab monotherapy group were better than those of the placebo group, with the monotherapy group exhibiting 5.3 months and 5.6 %, and the placebo group displaying 4.1 months and 1.9 %, respectively [13]. The KEYNOTE-059 clinical trial evaluated the safety and efficacy of pembrolizumab in 259 patients with previously treated GC or GEJC. The results showed an objective response rate of 11.6 % and a median duration of response of 8.4 months [14]. Similarly, in the phase III KEYNOTE-061 trial, pembrolizumab monotherapy for PD-L1 resulted in a Combined Positive Score (CPS) ≥5 for patients with advanced GC or gastroesophageal junction cancer (GEJC) that significantly prolonged the median overall survival (OS) compared to paclitaxel alone (10.4 months vs 8.3 months, respectively), and in patients with PD-L1 CPS≥10, the 24-month survival rate was significantly higher (32.1 % vs 10.9 %) [15]. However, in the ORIENT-16 phase III clinical trial, monotherapy with avelumab did not improve OS or progression-free survival (PFS) compared to chemotherapy in patients with GC or GEJC [16]. JAVELIN Gastric 100, another phase III clinical trial, showed that avelumab monotherapy had better OS for locally advanced GC or GEJC compared to chemotherapy [17].

**Table 1:** Clinical trials on PD-1/L1 inhibitors for GC.

Trial	Line	Phase	Drugs	Actual enrollment	Mian result	Reference
NCT02267343	third or later	Ⅲ	ONO-4538 vs Placebo	493	mOS: 5.26 Mos, mPFS: 1.61 Mos, ORR: 11.2 %	[13]
NCT02335411	second or later	Ⅱ	Pembrolizumab vs Pembrolizumab + Cisplatin+5-FU	318	mOS: 5.6 Mos, mPFS: 2 Mos, ORR: 11.6 %	[14]
NCT02370498	second	Ⅲ	Pembrolizumab vs Paclitaxel	592	mOS: 9.1 Mos, mPFS: 1.5 Mos (PD-L1 CPS≥1)	[15]
NCT02625623	third	Ⅲ	Avelumab vs Placebo	371	mOS: 4.6 Mos, mPFS: 1.4 Mos, ORR: 2.2 %.	[16]
NCT02625610	first	Ⅲ	Avelumab vs Oxaliplatin + Fluoropyrimidine	499	mOS: 10.4 Mos, mPFS: 3.2 Mos	[17]
NCT02746796	first	Ⅱ/Ⅲ	ONO-4538 vs SOX vs CapeOX	680	mOS: 17.45 Mos, mPFS: 10.45 Mos, ORR: 57 %	[76]
NCT03472365	first	Ⅱ	SHR-1210+ Capecitabine + Oxaliplatin vs SHR-1210 + Apatinib	67	mOS: 14.9 Mos, mPFS: 6.8 Mos, ORR: 58.3 %	[77]
NCT02954536	first	Ⅱ	Pembrolizumab + Trastuzumab + Chemotherapy	37	mOS: 27.3 Mos, mPFS: 13 Mos, ORR: 91 %	[78]
NCT03615326	first	Ⅲ	Pembrolizumab + Trastuzumab + Chemotherapy vs Placebo + Trastuzumab + Chemotherapy	732	ORR: 74.4 %	[38]
NCT02689284	first or later	Ib/2	Retifanlimab/Tebotelimab + Margetuximab + Chemotherapy	95	mOS: 12.48 Mos, mPFS: 2.73 Mos, ORR: 18.48 %	[79]
NCT03755440	first or second	Ⅱ	SHR-1210	6	mOS: 6.8 Mos, mPFS: 2.2 Mos, DCR: 66.7 %	[80]
NCT04890392	first	Ⅱ	Tislelizumab + Oxaliplatin	20	pCR: 25 %, MPR: 53.1 %	[81]
NCT02901301	first	Ib/II	Pembrolizumab + Trastuzumab + Chemotherapy	41	mOS: 19.3 Mos, mPFS: 8.6 Mos, DCR: 76.7 %	[82]
NCT03675737	first	Ⅲ	Pembrolizumab + Phemotherapy vs Placebo + Chemotherapy	1579	mOS: 12.9 Mos	[25]
NCT03609359	first or second	Ⅰ/Ⅱ	Lenvatinib vs Pembrolizumab	29	ORR: 69 %	[42]
NCT03019588	second	Ⅲ	Pembrolizumab vs Paclitaxel	94	mOS: 8 Mos, mPFS: 2 Mos, ORR: 13 %	[83]
NCT03745170	first	Ⅲ	Sintilimab + XELOX vs Placebo + XELOX	650	mOS: 15.2 Mos	[84]
NCT02872116	first	Ⅲ	Nivolumab + XELOX/FOLFOX vs XELOX/FOLFOX	2031	mOS: 14.3 Mos, mPFS: 8.3 Mos, ORR: 66 %	[47]
NCT02862535	first or later	Ib	Nivolumab vs Nivolumab + Andecaliximab	36	mPFS: 4.6 Mos, ORR: 50 %	[85]
NCT03710265	first	Ⅰ	SHR-1701	206	ORR: 20 %	[86]
NCT04345783	second	Ⅱ	Camrelizumab + Apatinib + S-1	30	mPFS: 6.5 Mos, ORR: 29.2 %	[87]
NCT04195828	first	Ⅱ	Camrelizumab + Apatinib + nab-paclitaxel + S-1 vs nab-paclitaxel + S-1	53	MPR: 33.3 %, ORR: 66.0 %	[88]

7FU^__^Fluorouracil; SOX^__^S-1/Oxaliplatin; CapeOX^__^Capecitabine/Oxaliplatin; XELOX^__^Capecitabine/Oxaliplatin; FOLFOX^__^5-Fluorouracil/Oxaliplatin; Mos^__^months; mOS^__^medium Overall Survival; mPFS^__^medium Progression-Free Survival; ORR^__^Overall Response Rate; pCR^__^pathologic Complete Response; MPR^__^Major Pathological Response.

### 3.2 PD-L1/PD-1 inhibitor combination therapy

The utilisation of PD-1/PD-L1 inhibitors in managing advanced GC or GEJC has, to a certain degree, enhanced patient prognosis and increased survival rates. However, because of the negative PD-L1 status of many patients and the limitations of immunotherapy monotherapy, current clinical research is more focused on exploring combinations with other treatment strategies. Existing studies show that combining PD-1/PD-L1 inhibitors with chemotherapy, radiotherapy, targeted drugs, and other ICIs can enhance anti-tumour effects.

#### 3.2.1 PD-L1/PD-1 inhibitors combined with surgery

Currently, surgery is the main treatment for GC, with D2 gastrectomy being one of the common surgical procedures. PD-1/PD-L1 inhibitors, as a form of immunotherapy, can be combined with surgery to improve treatment outcomes, although not all patients are suitable candidates. The ATTRACTION-5 trial, a large-scale, randomised, double-blind study, investigated the effectiveness of nivolumab (Opdivo), a PD-1 inhibitor, in combination with standard adjuvant chemotherapy compared to placebo plus chemotherapy for patients with stage III GE or GEJC after curative surgery with D2 lymph node dissection. The study did not find a statistically significant improvement in disease-free survival (DFS), a crucial measure of recurrence risk, in the nivolumab group compared to the placebo group. OS data may be available in future publications, but the initial results suggest limited benefit from adding nivolumab to the standard adjuvant treatment regimen. These findings suggest that nivolumab may not be a broadly effective adjuvant therapy for all patients with stage III GC or GEJC [18]. In a multicentre, open-label, single-arm Phase I clinical trial (JapicCTI-183895), investigators evaluated the safety and efficacy of neoadjuvant nivolumab monotherapy. The major pathological response (MPR) rate was 16 %, with a tumour reduction of at least 50 %, and where 90 % of patients achieved R0 resection. Notably, MPR was higher in patients with positive PD-L1 expression, high microsatellite instability (MSI), and/or high tumour mutational burden (TMB). This indicates that neoadjuvant nivolumab monotherapy is a feasible and safe treatment option for resectable gastric cancer, especially in patients with PD-L1 positive tumours, high MSI, or high TMB [19]. A prospective, two-arm Phase II study (NCT05844371) explored the effectiveness and safety of combining Tislelizumab, a PD-1 inhibitor, with the XELOX chemotherapy regimen (capecitabine and oxaliplatin) compared to chemotherapy alone in patients with lymph node-positive GC following D2 radical gastrectomy. The 1-year DFS rate in the Tislelizumab plus chemotherapy group (81.82 %) was higher compared with that of the chemotherapy alone group (71.43 %). However, the incidence of severe (grade 3–4) adverse events such as hypothyroidism and thrombocytopenia was significantly higher in the combination therapy group. The combination therapy of Tislelizumab and chemotherapy showed a promising trend in improving DFS in postoperative patients with GC with acceptable safety [20]. However, due to the lack of statistical significance in DFS improvement, further studies with larger sample sizes and longer follow-up periods are warranted to validate these findings.

In addition to improving efficacy and extending survival, combined immunotherapy can effectively convert unresectable GC into resectable disease, thereby enhancing treatment outcomes and prolonging survival. In a single-centre, open-label, single-arm Phase II trial, combination therapy resulted in a pathological complete response (pCR) in 9.1 % of patients and nearly pCR in 20.0 % of patients, indicating significant tumour burden reduction before surgery. Further, the safety of PD-1 inhibitors in adjuvant therapy is manageable, with adverse effects consistent with those observed in other clinical settings. The results of this study suggested that the combination of PD-1 inhibitors and mFOLFOX6 is effective in treating locally advanced GC, helping to reduce tumour burden before surgery [21]. Additionally, a case report has described a patient with locally advanced GC who underwent neoadjuvant treatment with a PD-1 blocker combined with chemoradiotherapy. Post-treatment, the previously unresectable tumour was significantly reduced and successfully resected. The patient did not experience severe immune-related adverse events (irAEs) during follow-up, and the survival period was extended [22]. This case indicated that PD-1 blocker combined with surgery may be a promising treatment option, potentially improving outcomes for patients with locally advanced GC.

#### 3.2.2 PD-L1/PD-1 inhibitors combined with chemotherapy

Chemotherapeutic agents are characterised by immunomodulatory properties and may exert a synergistic effect with PD-1/PD-L1 inhibitors, weakening cancer-related inhibition [23]. Additionally, chemotherapy can shrink tumours to reduce the risk of resistance to PD-1/PD-L1 inhibitors. Woosook et al. used oxaliplatin and 5-fluorouracil in a murine model of GC to show that 5-fluorouracil and oxaliplatin may reduce the number of myeloid-derived suppressor cells (MDSC), synergise with pembrolizumab, and promote CD8^+^ T cell tumour infiltration [24]. The KEYNOTE-859 study also showed that the pembrolizumab plus chemotherapy group had a better anti-tumour effect, and OS, while safety levels were significantly improved compared with the placebo plus chemotherapy group [25]. The Checkmate 649 clinical trial assessed the effectiveness of combining nivolumab with chemotherapy versus using chemotherapy alone in treating advanced GC. In patients with PD-L1 CPS ≥5, the combination of nivolumab and chemotherapy significantly improved median OS from 11.1 months to 14.4 months compared with chemotherapy alone, representing a 29 % reduction in the risk of death. Median PFS was also significantly prolonged from 6 months to 7.7 months, with a 32 % reduction in the risk of disease progression or death. The objective response rate (ORR) was 60 %, being substantially higher than the chemotherapy-alone arm. In the overall population, combination therapy also demonstrated a significant survival benefit, with median OS improving from 11.6 months to 13.8 months, representing a 20 % reduction in the risk of death. Similarly, Cindilizumab, when used alongside a regimen of albumin-bound paclitaxel, oxaliplatin, and S-1 chemotherapy (known as SAPO-S1 therapy for locally advanced gastric cancer), demonstrated effective therapeutic outcomes, reduced mortality risk, and minimal side effects [26,27]. The GEMSTONE-303 study demonstrated that, in patients with advanced GC or gastroesophageal junction adenocarcinoma and PD-L1 expression ≥5 %, the combination of sugemalimab and CAPOX significantly prolonged median PFS (7.6 months vs. 6.1 months) and OS (15.6 months vs. 12.6 months) compared to placebo. In terms of safety, the incidence of grade 3–5 drug-related adverse events was similar between the sugemalimab and CAPOX group and the placebo group, and no new safety signals were identified. These results suggest that the combination of sugemalimab and CAPOX can provide a new, effective, and well-tolerated first-line treatment option for patients with PD-L1-positive advanced GC [28]. The ORIENT-16 study confirmed that the combination of PD-1 monoclonal antibody and chemotherapy significantly improved survival in patients with unresectable locally advanced, recurrent, or metastatic GC or gastroesophageal junction adenocarcinoma. The combination therapy significantly reduced the risk of death in both the PD-L1 CPS ≥5 population and the overall population, with the median OS value increasing from 12.9 months to 18.4 months in the CPS ≥5 population and from 12.3 months to 15.2 months, respectively in the overall population. Consistent benefits were observed in all subgroup analyses. The success of the ORIENT-16 study has provided new medical evidence for first-line treatment options for patients with unresectable GC [29]. The RATIONALE-305 study, a global multicentre trial, demonstrated that the combination of camrelizumab and chemotherapy showed significant clinical benefits in patients with advanced GC, with a median OS of 15 months. Consistent survival benefits were observed in subgroup analyses across different populations and metastatic sites. Nearly one-third of responding patients experienced durable responses lasting more than 2 years, suggesting that this combination regimen offers a durable and significant clinical benefit. These findings support the use of camrelizumab in combination with chemotherapy to help patients with advanced GC achieve long-term survival [30]. The NEOSUMMIT-01 study contrasted perioperative chemotherapy with and without the PD-1 inhibitor toripalimab. Results showed a higher rate of pathological complete response (TRG 0) or near-complete response (TRG 1) in patients receiving toripalimab plus chemotherapy (44.4 %) compared to chemotherapy alone (20.4 %). Importantly, the addition of toripalimab to neoadjuvant chemotherapy did not elevate the risk of perioperative adverse events [31]. These findings underscore that patients with advanced GC can benefit from combining PD-1 inhibitors with chemotherapy, though further validation through larger-scale clinical trials is necessary.

#### 3.2.3 PD-L1/PD-1 inhibitors combined with radiotherapy

Radiotherapy is a method that can precisely kill tumour cells locally, and it can also activate immunogenic cell signalling pathways, enhancing the immune response to eliminate tumour cells [32]. Existing research has elucidated that anti-PD-L1 therapy amplifies the effectiveness of ionising radiation (IR) through a mechanism reliant on cytotoxic T cells using a murine model of forestomach carcinoma. The combined use of IR and anti-PD-L1 stimulates CD8^+^ T-cell responses and reduces the local concentration of MDSCs within the tumour via tumour necrosis factor (TNF), thereby enhancing the immune microenvironment of the tumour and consequently producing anti-tumour effects [33]. These findings provide an important foundation for combining PD-1/PD-L1 inhibitor therapy with radiotherapy in the treatment of cancer. At the cellular level, ablative hypofractionated radiation therapy (AHFRT) reduces local accumulation of MDSCs, decreases PD-1 expression, and thus activates CD8^+^ T cell immune response in mice [34]. When AHFRT is combined with anti-PD-L1, AHFRT shows more significant effects in the treatment of patients with GC. This combined therapy strategy demonstrates the potential for synergistic effects and provides strong evidence for more effectively addressing tumour challenges. Current trials are investigating the efficacy and safety of PD-1 in combined radiotherapy strategies. A Phase I/II study evaluating the efficacy of nivolumab combined with radiotherapy versus monotherapy as a maintenance treatment for advanced GC is currently underway, with subsequent results eagerly anticipated [35].

#### 3.2.4 PD-L1/PD-1 inhibitors combined with targeted therapy

Trastuzumab, in combination with chemotherapy, serves as the standard first-line treatment for HER2-positive advanced GC or GEJC. Trastuzumab, on the one hand, blocks intracellular HER2 signalling and inhibits cancer cell growth by acting on the extracellular domain of HER2, and on the other hand, activates the anti-tumour activity of the host immune system. In preclinical models, combining anti-HER2 therapy with PD-1/PD-L1 inhibitors helps to exert a synergistic anti-tumour effect [36,37]. The PANTHERA trial demonstrated the efficacy of pembrolizumab combined with trastuzumab and chemotherapy as a first-line treatment for HER-2 positive advanced GC, with an ORR of 76.7 %, DCR of 97.7 %, median OS of 19.3 months, and 1-year OS rate of 80.1 %. Adding pembrolizumab to the trastuzumab and chemotherapy regimen can significantly inhibit tumour growth, with some patients even achieving complete remission, while markedly improving the objective response rate in patients. The preliminary results of the KEYNOTE-811 study suggested that the combination therapy of pembrolizumab, trastuzumab, and chemotherapy had the potential to become a new standard treatment for HER2-positive gastric adenocarcinoma or gastroesophageal junction adenocarcinoma [38]. A phase III clinical trial showed that pembrolizumab combined with trastuzumab and chemotherapy as a first-line treatment for HER-2 positive GC achieved an ORR of 74.4 %, a 22.7 % increase compared to the placebo group, with a complete response rate of 11 % (compared to 3 % in the control group) [39]. These data indicated the robust therapeutic effect of PD-1/PD-L1 inhibitors combined with Her-2 targeted therapy.

Anti-angiogenic drugs target vascular endothelial growth factor (VEGF) and its receptor pathway to reduce angiogenesis, regulate the tumour microenvironment, and thus exert anti-tumour effects, and combined with immunotherapy drugs can have a synergistic anti-tumour effect. Niu et al. showed that combining a novel anti-TGF-β/VEGF bispecific antibody Y332D with PD-1 blockade increased the density and function of tumour-infiltrating lymphocytes, showing a stronger anti-tumour effect [40]. According to preclinical data, the bispecific antibody HB0025 was more effective in inhibiting cancer growth than using the parent anti-PD-L1mAb or VEGFR1D2 fusion protein alone, and HB0025 more effectively inhibited cancer growth, benefiting more clinical patients [41]. Lenvatinib, a multi-kinase inhibitor targeting VEGFR1-3, FGFR1-4, RET, KIT, and PDGFR, demonstrated good anti-cancer activity in patients with advanced GC, with an ORR of 69 % according to clinical trial NCT03609359 [42]. Ramucirumab, a VEGFR-2 antagonist, based on a phase I/II clinical study (NCT02999295), combined with nivolumab and paclitaxel as second-line treatments for advanced GC, showed a mOS of 13.1 months, ORR of 37.2 %, and a 6-month PFS rate of 46.5 %, significantly benefiting patients with advanced GC [43].

Small molecule inhibitors are closely related to PD-1/L1, and research on related combination therapies is booming. The utilisation of small molecule inhibitors in conjunction with anti-PD-1/PD-L1 drugs encompasses a range of options, including EGFR inhibitors, PI3K and MAPK pathway inhibitors, poly ADP ribose polymerase inhibitors, cyclin-dependent kinase inhibitors, DNA methyltransferase inhibitors, indoleamine 2,3-dioxygenase 1 inhibitors, adenosine (A)/adenosine receptor and their antagonists should be a major research trend in the future.

#### 3.2.5 PD-L1/PD-1 inhibitors combined with other ICIs

In addition to PD-1, T cells express other immune checkpoint receptors on their surface, such as TIM-3, LAG-3, and 2B4. In current clinical research, the most attention is focused on the combination of ICIs, specifically the concurrent use of cytotoxic T-lymphocyte associated protein 4 (CTLA-4) antibodies and PD-1/PD-L1 antibodies. CTLA-4 inhibitors primarily function by obstructing the transmission of inhibitory signals, effectively releasing the brakes on T cell activation and thereby maintaining the activated state of T cells. Consequently, CTLA-4 primarily plays a role in regulating the transmission of inter-lymphocyte interaction signals. On the other hand, PD-1 antibodies primarily inhibit the activation process of the immune response, while PD-L1 serves as a crucial immune molecule involved in the activation of immune cells within tumour cells and the tumour microenvironment. Therefore, the combined application of PD-1 and CTLA-4 inhibitors can lead to synergistic effects, as they simultaneously target different levels of immune regulatory mechanisms [44]. Therefore, the combined application of PD-1 and CTLA-4 inhibitors can produce synergistic effects, simultaneously acting on different levels of immune regulatory mechanisms.

Ipilimumab is an anti-CTLA-4 antibody, expressed on the surface of T cells and was the first inhibitory receptor target used in clinical research. The results of the CheckMate 649 phase III clinical trial showed that in patients with PD-L1 CPS ≥5, nivolumab plus ipilimumab had a better remission rate compared to chemotherapy, but its PFS and ORR were not improved [45]. A phase II GERCOR NEONIPIGA study assessed the pCR rate of perioperative nivolumab and ipilimumab combined treatment in resectable locally advanced dMMR/MSI-H gastric/GEJ adenocarcinoma patients. Of the 29 patients who underwent R0 resection, the pCR rate was 58.6 %, with no patients relapsing, suggesting that neoadjuvant treatment based on nivolumab and ipilimumab benefits patients [46]. Currently, a phase III clinical study (CheckMate 649) on the combination of nivolumab and ipilimumab is ongoing, expected to bring new breakthroughs in the first-line treatment of advanced GC [47].

This combined application is expected to more comprehensively activate the immune system and enhance the immune response to tumours. Previous studies have shown that compared to single-agent PD-1/PD-L1 inhibitor therapy, the combination of multiple immune checkpoint receptor antibodies, such as LAG-3 inhibitors [48], TIGIT inhibitors [49], etc., significantly improved anti-tumour efficacy. However, this combination therapy may increase the risk of adverse effects, requiring more research to address its potential challenges and uncertainties.

## 4 Adverse reactions of PD-L1/PD-1 inhibitors in GC treatment

Immunotherapy can overactivate the immune system, causing irAEs. Currently, adverse reactions remain a major challenge in using PD-1/PD-L1 inhibitors. These inhibitors may cause irAEs by de-repressing T cell functions, but their specific pathophysiological mechanisms are not fully elucidated yet. A meta-analysis including 35 studies and 8,370 cases reported that the incidence of irAEs in patients treated with PD-1/PD-L1 inhibitors was 17.1 %, with 4 % of 4,921 patients experiencing grade ≥3 irAEs [50]. irAEs involve multiple organ systems. Mild cases may include skin reactions like rash, itchiness, minor gastrointestinal discomfort such as mild diarrhoea, loss of appetite, and flu-like symptoms like fatigue, headache, and muscle pain. However, some patients may experience severe irAEs, with skin reactions escalating to severe rashes or allergic reactions. Gastrointestinal reactions can lead to severe diarrhoea and inflammation. Liver function abnormalities may cause symptoms like jaundice. In rare cases, fatal irAEs can occur, including severe colitis, pneumonia, hepatitis, encephalitis, myocarditis, adrenal insufficiency, and diabetic ketoacidosis. A preclinical murine model showed that using anti-PD-1 monoclonal antibody alone induced myocarditis in A/J mice [51].

When using PD-1/PD-L1 inhibitors, it is essential to closely monitor their anti-tumour effects and cautiously address the potential occurrence of irAEs. Future research should focus on developing new formulations to enhance efficacy and establish grading and treatment strategies for adverse reactions. Additionally, standardising the preparation, quality control, and clinical application standards of PD-1 inhibitors is crucial to reduce adverse reactions and support the widespread clinical use of PD-1 immunotherapy.

## 5 Potential biomarkers for PD-L1/PD-1 inhibitor treatment in GC

ICIs represented by PD-1/PD-L1 inhibitors can disrupt the immunosuppressive microenvironment of the tumour, restoring the ability of the immune cell to recognise and kill cancer cells, thereby effectively improving the prognosis of patients with GC. However, PD-1/PD-L1 inhibitor therapy only benefits a subset of patients with GC, making it crucial to identify patient subgroups more likely to benefit from immunotherapy for precision treatment.

CPS for PD-L1, MSI status, TMB, and gene expression profiling are recommended as common biomarkers for GC [52]. In the CheckMate 032 clinical trial, patients with GC showing higher PD-L1 expression by CPS gained the most clinical benefit, suggesting that PD-L1 expression is a potential factor in predicting clinical efficacy [53]. A meta-analysis showed that CPS is superior to TPS in scoring methods and thresholds for PD-L1 expression, confirming the predictive value of PD-L1 expression for assessing an ICI response [54]. Additionally, two meta-analysis results suggested that in the subgroup of patients with PD-L1 CPS ≥10, immunotherapy significantly benefited the tumour response rate. CPS ≥10 has the potential to serve as a precise marker for the population that could benefit from combined immunotherapy [55,56]. Three clinical trials, KEYNOTE-059, KEYNOTE-061, and KEYNOTE-062 assessed the anti-tumour activity of pembrolizumab versus chemotherapy in patients with MSI-H advanced GC or GEJC. In these trials, MSI-H tumour patients treated with pembrolizumab or pembrolizumab combined with chemotherapy did not reach median overall survival, indicating MSI-H status as a potential biomarker for PD-1/PD-L1 inhibitor efficacy in advanced GC or GEJC. The phase III KEYNOTE-062 study [57] and KEYNOTE-061 trial [58] on treating GC or GEJ adenocarcinoma reported consistent results, with high TMB found to be associated with better prognosis, suggesting TMB-H status as having clinical utility in predicting the response of patients with CG to PD-1 inhibitors. Some studies have shown that patients with GC related to Epstein-Barr virus exhibit significant clinical efficacy when receiving immunotherapy, with EBV being a predictive biomarker for the efficacy of PD-1/PD-L1 inhibitors [59,60]. Besides, many studies have evaluated other predictive biomarkers for GC or GEJC immunotherapy (Table 2).

**Table 2:** The potential biomarkers for PD-1/PD-L1 inhibitor in advanced GC.

Author	n	Year	Therapeutic Drugs	Biomarker	Ref
Gu	416	2023	PD-1 Inhibitors	ARID1A	[61]
Ding	30	2022	PD-1 Inhibitors	SII and PNI	[62]
Kawakami	439	2023	PD-1 Inhibitors	sPD-L1 and GPS	[63]
Schoemig-Markiefka	56	2021	PD-1/PD-L1 Inhibitors	PD-L1 CPS	[64]
Jia	80	2022	PD-1/PD-L1 Inhibitors	CLDN18.2	[65]
Li	50	2023	PD-1 Inhibitor	GPC3	[66]
Zhang	27	2023	PD-1 Inhibitor	MFSD2A	[67]
Nose	29	2023	PD-1 Inhibitor	CD103	[68]
Catenacci	86	2023	PD-1 Inhibitor	ctDNA	[69]
Kurosaki	20	2023	PD-1 Inhibitor	sPD-1 and sPD-L1	[70]
Qi	52	2023	PD-1 Inhibitors/Chemotherapy	IL-4 and IL-6	[71]
Chida	36	2022	PD-1 Inhibitor	MSI-H/dMMR	[72]
Fan	50	2021	PD-1 Inhibitor	NLR, MLR and PLR	[73]
Lu	78	2022	PD-1 Inhibitor	PD-L1 and HA	[74]
Jiang	100	2023	PD-1 Inhibitor	CD36-BATF2\MYB	[75]

ARID1A^__^AT-Rich Interaction Domain 1A; SII^__^Systemic Immune-Inflammatory Index; PNI^__^ Prognostic Nutritional Index; sPD-L1^__^Soluble Forms of Programmed Cell Death Protein Ligand 1; CPS^__^IHC Combined Positive Score; CLDN18.2^__^Isoform 2 of Claudin-18; GPC3^__^Glypican-3; MFSD2A^__^Major Facilitator Superfamily Domain Containing 2A; CD103^__^Integrin Alpha-E; ctDNA^__^Circulating Tumour DNA (ctDNA); sPD-1^__^Soluble Forms of Programmed Cell Death Protein 1; IL-4^__^Interleukin 4; IL-6^__^Interleukin 6; MSI-H^__^Inmicrosatellite Instability-High; dMMR^__^Mismatch Repair-deficient; NLR^__^Neutrophil-to-Lymphocyte Ratio; MLR^__^Monocyte -to-Lymphocyte Ratio; PLR^__^Platelet-to-Lymphocyte Ratio; HA^__^Hyperamplifcation; CD36^__^CD36 Antigen (Collagen Type I Receptor, Thrombospondin Receptor); BATF2^__^Basic Leucine Zipper ATF-Like Transcription Factor 2; MYB^__^Myeloblastosis Family of Transcription Factor.

Currently, exploring predictive biomarkers in clinical research faces several limitations, such as small treatment sample sizes, inconsistent research methods, potential false-negative results, and high testing costs. To ensure the reliability of these biomarkers, conducting high-quality and standardised clinical research is necessary. Moreover, the current ability of a single biomarker to predict tumour treatment efficacy is quite limited. Therefore, developing a comprehensive assessment model combining multiple biomarkers and constructing corresponding predictive models will more effectively identify patient groups best suited for immunotherapy and optimise treatment plans.

## 6 Summary and outlook

In recent years, significant advances have been made in the application of PD-1/PD-L1 inhibitors for the treatment of advanced gastric cancer. These advancements underscore the potential of immunotherapy to change the landscape of cancer treatment, offering new hope where traditional therapies have plateaued. Despite the encouraging outcomes, challenges remain, particularly in optimizing the efficacy of these inhibitors and managing associated adverse events. One of the most promising aspects of PD-1/PD-L1 inhibitors is their ability to reinvigorate the response of the immune system to cancer. Clinical trials have demonstrated that these inhibitors can significantly improve the objective response rates and overall survival of patients with advanced gastric cancer. However, not all patients respond equally to PD-1/PD-L1 monotherapy, highlighting the need for biomarkers that can predict which patients are most likely to benefit from these treatments.

The combination of PD-1/PD-L1 inhibitor usage with other treatment modalities such as chemotherapy, radiotherapy, and targeted therapies has shown enhanced efficacy compared to monotherapy. This approach leverages the strengths of each treatment modality, providing a synergistic effect that improves patient outcomes. Future research should continue to explore these combination therapies to establish optimal treatment protocols and identify patient subgroups that may benefit the most from these multi-pronged approaches. Safety remains a critical concern with PD-1/PD-L1 inhibitors, since irAEs can be severe. Managing these adverse events requires a comprehensive understanding of the underlying mechanisms and the development of strategies to mitigate risks. Continued monitoring and reporting of adverse events in clinical trials will be crucial in refining safety guidelines.

Looking forward, the discovery of reliable predictive biomarkers is paramount. Biomarkers such as PD-L1 expression levels, microsatellite instability, and tumour mutational burden have shown potential in predicting responses to immunotherapy. Further research is needed to validate these biomarkers and integrate them into clinical practice to tailor treatments to individual patient needs effectively. In summary, the integration of PD-1/PD-L1 inhibitors into the therapeutic arsenal against advanced gastric cancer marks a significant advancement in oncology. While challenges remain, the progress made thus far provides a solid foundation for future research and clinical application. Continued efforts via clinical trials, biomarker discovery, and combination therapy optimisation will pave the way for more effective and personalised treatment strategies, ultimately improving outcomes for patients with advanced gastric cancer.

## 7 Conclusions

Currently, immunotherapy is a trending topic in the field of cancer treatment and is an important therapy alongside radiotherapy, chemotherapy and conventional surgery for the treatment of tumours. Among these various approaches, PD-1/PD-L1 immunotherapy stands out for its ability to boost the immune response of the patient against tumour cells. To date, PD-1/PD-L1 combination therapy has demonstrated robust clinical efficacy in managing various cancers, including non-small cell lung cancer, colorectal cancer, gastric cancer, breast cancer, and liver cancer, where it can work synergistically with radiotherapy, chemotherapy, and targeted treatments to combat tumours.

However, our current understanding represents merely the tip of the iceberg, particularly in the field of gastric cancer treatment, where the application of PD-1/PD-L1 inhibitors is confronted by numerous key scientific challenges. Among these, managing irAEs brought about by immunotherapy remains a critical issue. The toxic effects associated with combination therapy highlight the necessity to further refine the combined medication regimens, dosages, and optimal sequencing to achieve the best treatment outcomes. Additionally, effective prevention or reduction in the incidence of irAEs, as well as the development and implementation of comprehensive assessment and predictive models involving multiple biomarkers, are urgent matters that demand in-depth exploration. A significant amount of basic and clinical research is still required to provide insights for resolving these challenges, thereby advancing the immunotherapy approaches in gastric cancer treatment.

## Data availability statement

The authors confirm that the data supporting the findings of this study are available within the article. For additional information or specific requests related to the data, please contact the corresponding author.

## Funding

This research was funded by the 10.13039/501100001809National Natural Science Foundation of China (Grant Number 81773538), Project 333 of Jiang Su Province (Grant number S333YQ002) and Jiangsu Provincial Medical Key Discipline Cultivation Unit (Grant number JSDW202233).

## CRediT authorship contribution statement

**Jin Shi:** Writing – review & editing, Conceptualization. **Xudong Song:** Writing – original draft. **Zihao Gao:** Writing – original draft. **Dezhu Dai:** Writing – original draft. **Fan Ding:** Writing – original draft. **Xu Wu:** Writing – review & editing. **Wufei Dai:** Writing – review & editing. **Guoquan Tao:** Writing – review & editing, Funding acquisition, Conceptualization.

## Declaration of competing interest

The authors declare that they have no known competing financial interests or personal relationships that could have appeared to influence the work reported in this paper.
